# Characterization of Post-Translational Modifications to Calsequestrins of Cardiac and Skeletal Muscle

**DOI:** 10.3390/ijms17091539

**Published:** 2016-09-13

**Authors:** Kevin M. Lewis, Gerhard R. Munske, Samuel S. Byrd, Jeehoon Kang, Hyun-Jai Cho, Eduardo Ríos, ChulHee Kang

**Affiliations:** 1 Department of Chemistry, Washington State University, Pullman, WA 99164, USA; kevin_lewis@wsu.edu; 2 School of Molecular Biosciences, Washington State University, Pullman, WA 99164, USA; munske@wsu.edu; 3 The Gene and Linda Voiland School of Chemical Engineering and Bioengineering, Washington State University, Pullman, WA 99164, USA; samuelsbyrd@comcast.net; 4 Division of Cardiology, Department of Internal Medicine, Seoul National University, Seoul 110-744, Korea; medikang@gmail.com (J.K.); hyunjaicho@snu.ac.kr (H.-J.C.); 5 Department of Molecular Biophysics and Physiology, Rush University, Chicago, IL 60612, USA; Eduardo_Rios@rush.edu

**Keywords:** calsequestrin, calcium, calcium-binding protein, glycosylation, phosphorylation, post-translational modification (PTM)

## Abstract

Calsequestrin is glycosylated and phosphorylated during its transit to its final destination in the junctional sarcoplasmic reticulum. To determine the significance and universal profile of these post-translational modifications to mammalian calsequestrin, we characterized, via mass spectrometry, the glycosylation and phosphorylation of skeletal muscle calsequestrin from cattle (*B. taurus*), lab mice (*M. musculus*) and lab rats (*R. norvegicus*) and cardiac muscle calsequestrin from cattle, lab rats and humans. On average, glycosylation of skeletal calsequestrin consisted of two *N*-acetylglucosamines and one mannose (GlcNAc_2_Man_1_), while cardiac calsequestrin had five additional mannoses (GlcNAc_2_Man_6_). Skeletal calsequestrin was not phosphorylated, while the C-terminal tails of cardiac calsequestrin contained between zero to two phosphoryls, indicating that phosphorylation of cardiac calsequestrin may be heterogeneous in vivo. Static light scattering experiments showed that the Ca^2+^-dependent polymerization capabilities of native bovine skeletal calsequestrin are enhanced, relative to the non-glycosylated, recombinant isoform, which our crystallographic studies suggest may be due to glycosylation providing a dynamic “guiderail”-like scaffold for calsequestrin polymerization. Glycosylation likely increases a polymerization/depolymerization response to changing Ca^2+^ concentrations, and proper glycosylation, in turn, guarantees both effective Ca^2+^ storage/buffering of the sarcoplasmic reticulum and localization of calsequestrin (Casq) at its target site.

## 1. Introduction

Calsequestrin (Casq) is considered to be the major Ca^2+^-storage/buffer protein within the sarcoplasmic reticulum (SR) [1] of both cardiac and skeletal muscle, where it resides as either its cardiac (Casq2) or skeletal (Casq1) isoform. Casq binds Ca^2+^ with a capacity roughly equivalent to a half of its total negative charge and an overall dissociation constant of ~1 mM [2,3]. Both Casq1 and Casq2 are highly conserved across all species, with sequence conservation being 92% for Casq1 and 85% for Casq2 among humans (*H. sapiens sapiens*), cattle (*B. taurus*, bovine), the common house mouse (*M. musculus*), European rabbits (*O. cuniculus*) and the common rat (*R. norvegicus*). The total Asp and Glu content is roughly equal between the two Casq isoforms, and the total Arg and Lys content is highly conserved, totaling 29 in Casq1 and 40 in Casq2 in various animals, which leads to Casq1 having a higher net negative charge than Casq2. In addition, the C-terminal tail of Casq2 contains non-acidic residues, such as Asn, Gly and Ser, while Casq1 contains only acidic residues (Asp and Glu).

When studied in solution, Casq polymerizes as free Ca^2+^ concentrations increase and depolymerizes as Ca^2+^ concentrations decrease. Those works in vitro suggest that Casq oligomers should be formed and dismantled in vivo, in response to Ca^2+^ entry into and release from the SR. When imaged in situ, in muscle cells at rest, structures consistent with the Casq isoforms exist in terminal cisternae (TC) of the SR. These structures have a strikingly different appearance in skeletal and cardiac muscle. In skeletal muscle, they consist of linear polymers that ramify to a three-dimensional dense network that fills the large TC of the skeletal myofibrils. Deep-etch electron microscopy images of the SR terminal cisternae in *Nerodia sipedon* show that the dense structures that fill the terminal cisternae are indeed linear Casq polymers [4] with branch points, endorsing the Casq polymer structure predicted by results in vitro [5]. In cardiac muscle instead, Casq2 forms clumps, denser and more spatially-confined within the shallow, pancake-like TC of cardiac myocytes. We have previously shown distinct Ca^2+^-dependent polymerization behavior unique to each of the two isoforms [6,7]. The difference in structural arrangement, however, appears to be related to the relative abundance of Casq vs. its membrane anchoring partners, triadin and junctin, rather than to any divergence in molecular structure, because the structural differences are minimized when Casq2 is experimentally overexpressed [8,9]. In summary, there is good evidence for polymerization of both Casq isoforms in situ. Evidence in vivo for depolymerization as SR Ca^2+^ concentrations decrease is instead indirect and has been communicated only recently [10].

The arrangement of calsequestrin in linear polymers, tethered to the SR junctional membrane and ending near the luminal mouth of Ca^2+^ release channels, is seen as having mechanistic significance. Ca^2+^ ions are proposed to diffuse along the surface of the linear Casq polymer, like current through a wire, guided by the steep Ca^2+^ gradient, ~10^4^, between the SR and cytoplasm. In an example of diffusion enhancement by the reduction of dimensionality [11], such a process would facilitate release flux, as diffusion would then proceed along a single dimension [5,6,12,13].

Casq undergoes post-translational modification during its transit to its final destination in the junctional SR [14,15]. Previous reports have characterized canine Casq1 and Casq2 as containing glycosyl chains with compositions of GlcNAc_2_Man_7-5_ and GlcNAc_2_Man_9-8_, respectively, as well as three phosphorylation sites uniquely in canine Casq2 [14,15]. We previously determined the specific site and degree of glycosylation in Casq1 using the crystal structure of rabbit Casq1 and mass spectrometry of its peptide fragments [16]. We also showed that both glycosylation and phosphorylation alter the physical characteristics of Casq substantially, including its oligomerization and Ca^2+^-binding capacity [16,17]. Based on those data, we proposed that both glycosylation [16] and phosphorylation [17] can effectively modulate not only Casq processing/targeting, but also its function, based on the physiological needs of the organism, and potentially serving as a mechanism for the regulation of Ca^2+^ homeostasis and signaling.

Considering the necessity of post-translational modifications for proper function and targeting of Casq, improper post-translational modifications could inhibit the protein’s in vivo functions, with resulting clinical manifestations. One of the diseases caused by dysfunctional Casq2 is catecholaminergic polymorphic ventricular tachycardia type 2 (CPVT2), a potentially lethal hereditary illness caused by mutations to the *Casq2* gene [18,19]. One of the CPVT-related mutations, K206N, leads to an additional N-linked glycosylation site on Casq, which inhibits proper dimer formation in solution [20] and is expected to impede the normal function of Casq in vivo. Because of the observed effects of the K206N mutation, it follows that improper post-translational modifications can produce pathological consequences. In addition, based on the symptomatic similarity between malignant hyperthermia (MH) and CPVT2 and the observation of MH susceptibility in Casq1-null mice, mutations to *Casq1*, such as M87T and D244G, have been identified as possible causes or enablers of malignant hyperthermia and other skeletal muscle diseases, like vacuolar aggregate myopathy and central core disease [21,22,23]. Thus, improper Casq1 glycosylation likely induces deleterious effects similar to those mutations.

A first step toward identifying the in vivo effect and pathological link for those post-translational modifications is to determine the exact pattern and effect of glycosylation and phosphorylation in humans and other model animal species. For this purpose, we have characterized the post-translational modifications of the rat and mouse model systems and have compared them to those of native human Casq2 obtained from cardiac muscle from two separate donors. In addition, we analyzed the post-translational modifications and their effects in bovine Casq1 and Casq2, as close models of the corresponding human proteins.

## 2. Results

### 2.1. Glycosylation and Phosphorylation of Casq1

In order to determine the exact profile of the post-translational modifications to mammalian Casq, we characterized, via mass spectrometry, the glycosylation and phosphorylation of Casq1 from cattle (*B. taurus*), lab mice (*M. musculus*) and lab rats (*R. norvegicus*) and Casq2 from cattle, lab rats and humans.

#### 2.1.1. Casq1 Glycosylation

**Figure 1:**
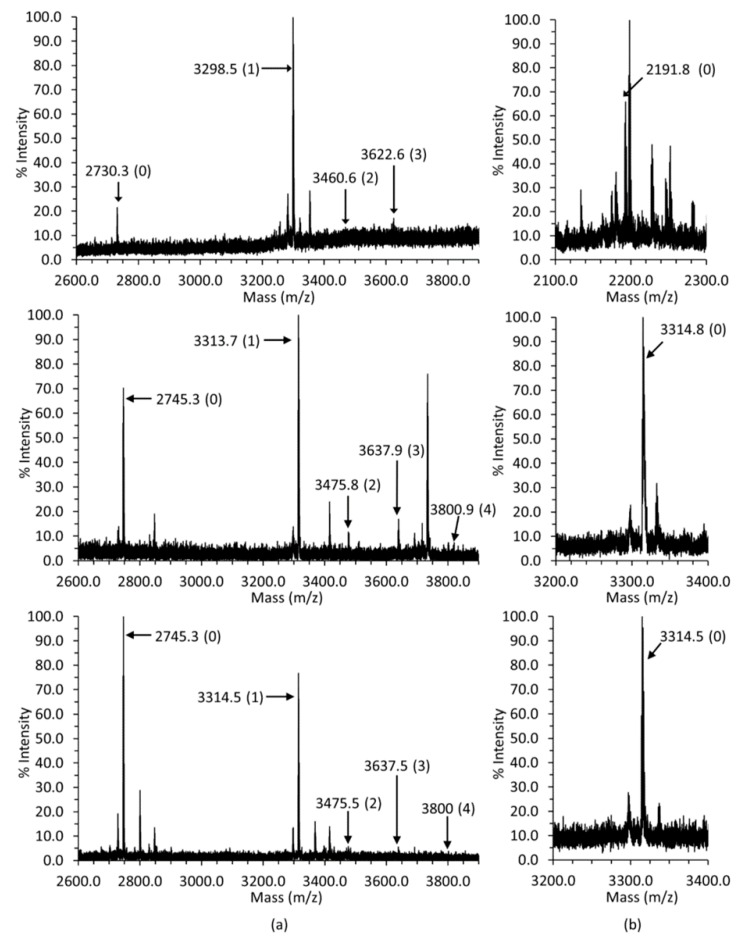
The mass spectra of bovine (**top**), mouse (**middle**) and rat Casq1 (**bottom**). (**a**) Calsequestrin 1 (Casq1) glycosylation spectra. The mass of each peak corresponding to either the non-glycosylated peptide or a glycopeptide is labeled, with each mannose contributing an extra 162 *m*/*z*. The parenthetical number, (*n*), associated with each mass indicates the number of mannose present, except the case of the non-glycosylated peptide, which does not have any sugars attached. The GlcNAc_2_ is implied, i.e., (*n*) denotes GlcNAc_2_Man_(*n*)_; (**b**) the C-terminal peptide masses. The parenthetical zero indicates no phosphoryls are present. The spectra for the peptides were collected using a 4800 MALDI TOF/TOF Analyzer (Applied Biosystems). All spectra were collected in negative reflector mode.

The major bovine, mouse and rat Casq1 glycoform was GlcNAc_2_Man_1_ (3298.5, 3313.7 and 3314.5 *m*/*z*, respectively) based on peak height, with the non-glycosylated peptides (2730.3, 2746.4 and 2745.3 *m*/*z*, respectively) also present (Figure 1a). The GlcNAc_2_Man_2-4_ glycoforms (successive +162 *m*/*z* peaks) were present in smaller amounts (Figure 1a) and were assigned as such if the peak corresponding to their expected mass had a signal-to-noise ratio greater than 1.0.

#### 2.1.2. Casq1 Phosphorylation

The high negative charge of the C-terminal peptide enabled its capture by the TiO_2_ + ZrO_2_ tip in a manner similar to phosphopeptides (Figure 1b). No peak, which indicates phosphorylation, 80 *m*/*z* heavier than the expected mass of unmodified bovine, mouse or rat Casq1 C-terminal peptides, was observed, informing that the Casq1 C-terminal tail is not phosphorylated.

### 2.2. Glycosylation and Phosphorylation of Casq2

#### 2.2.1. Casq2 Glycosylation

**Figure 2:**
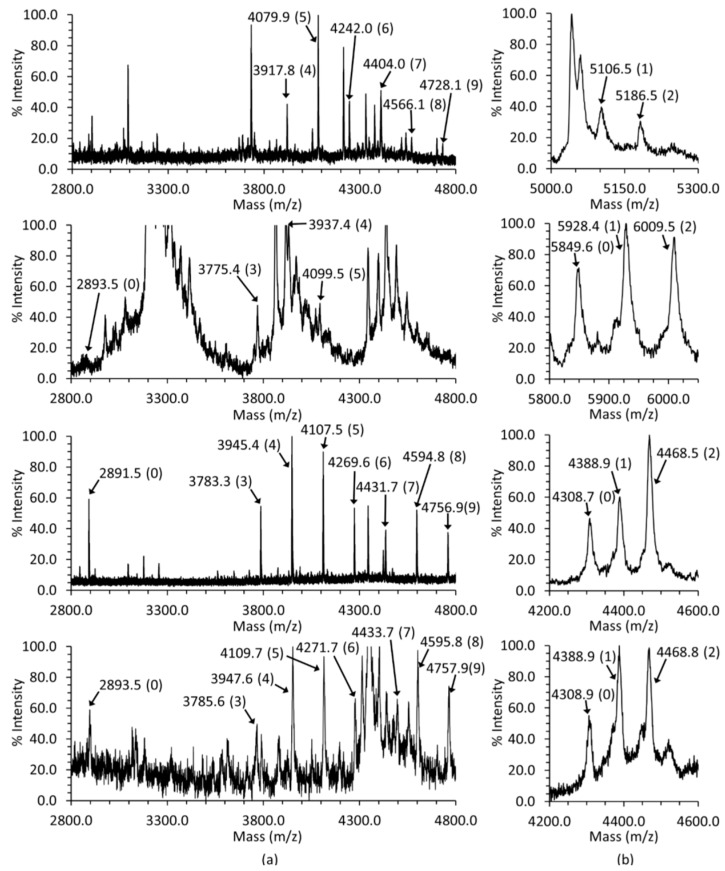
The mass spectra of bovine (**top** row), rat (**middle** row) and the two human Casq2s (**bottom** two rows). (**a**) Casq2 glycosylation spectra. The mass of each peak corresponding to either the non-glycosylated peptide or a glycopeptide is labeled, with each mannose contributing an extra 162 *m*/*z*. The parenthetical number, (*n*), associated with each mass indicates the number of mannose present, except the case of the non-glycosylated peptide, which does not have any sugars attached. The GlcNAc_2_ is implied, i.e., (*n*) denotes GlcNAc_2_Man_(*n*)_; (**b**) the C-terminal peptide masses with their corresponding number of phosphoryls are indicated in parentheses. A difference of 80 *m*/*z* indicates an additional phosphoryl group (as HPO_3_). The spectra for the peptides were collected using a 4800 MALDI TOF/TOF Analyzer (Applied Biosystems). The glycosylation mass spectra of bovine and the first human heart (first and third row, respectively) were collected in positive reflector mode, whereas the same spectra for rat and the second human heart (second and fourth row, respectively) were collected in positive linear mode. All Casq2 phosphorylation mass spectra were collected in linear positive mode.

Unlike the preference of Casq1 for GlcNAc_2_Man_1_, there did not appear to be any preferred extent of glycosylation for Casq2, aside from its apparent minimum of GlcNAc_2_Man_3-4_ as inferred by relative peak heights (Figure 2a). Bovine Casq2 glycosylation ranged from GlcNAc_2_Man_4_ (3917.8 *m*/*z*) to GlcNAcMan_9_ (4728.1 *m*/*z*), with no apparent non-glycosylated form. Rat Casq2 ranged from GlcNAc_2_Man_3_ (3775.4 *m*/*z*) to GlcNAc_2_Man_5_ (4099.5 *m*/*z*), with the non-glycosylated form present in very small amounts (3893.5 *m*/*z*). The two human Casq2s ranged from GlcNAc_2_Man_3_ (3783.8 *m*/*z* and 3785.6 *m*/*z*) to GlcNAc_2_Man_9_ (4758.8 and 4757.9 *m*/*z*), with the non-glycosylated form present in relatively large amounts for one of them (2891.5 *m*/*z*) and relatively small amounts (2893.5 *m*/*z*) for the other. The signal-to-noise ratios for most peaks were sufficient to correlate their experimental mass to their expected mass and distinguish them from peaks corresponding to other peptides. The non-glycosylated peptide of rat Casq2 was an exception, as it had a weak signal, but enough signal was present to distinguish it from the background and to calculate its mass from the data.

#### 2.2.2. Casq2 Phosphorylation

A mixture of non-phosphorylated, mono-phosphorylated and di-phosphorylated C-terminal peptides was present in all bovine, human and rat Casq2 mass spectra (Figure 2b). The non-phosphorylated C-terminal peptide was also present in the human and rat Casq2 spectra, suggesting that Casq2 exists simultaneously in non-phosphorylated, mono-phosphorylated and di-phosphorylated forms.

### 2.3. Casq1 Crystal Structures

To investigate any structural significance/role of glycans or structural change due to the presence of glycans, the crystal structures for both native and recombinant bovine Casq1, which correspond to the glycosylated and nonglycosylated form, respectively, were determined under the same condition. Two structures were closely compared in order to find the structural impact of glycosylation. Calsequestrin crystallized in two forms: the low-Ca^2+^ forms of native and recombinant bovine Casq1 crystallized in the absence of added Ca^2+^ in the crystallization buffer; the high-Ca^2+^ forms crystallized in the presence of 2.5 mM Ca^2+^ (17× molar excess of Ca^2+^ added to solution).

**Figure 3:**
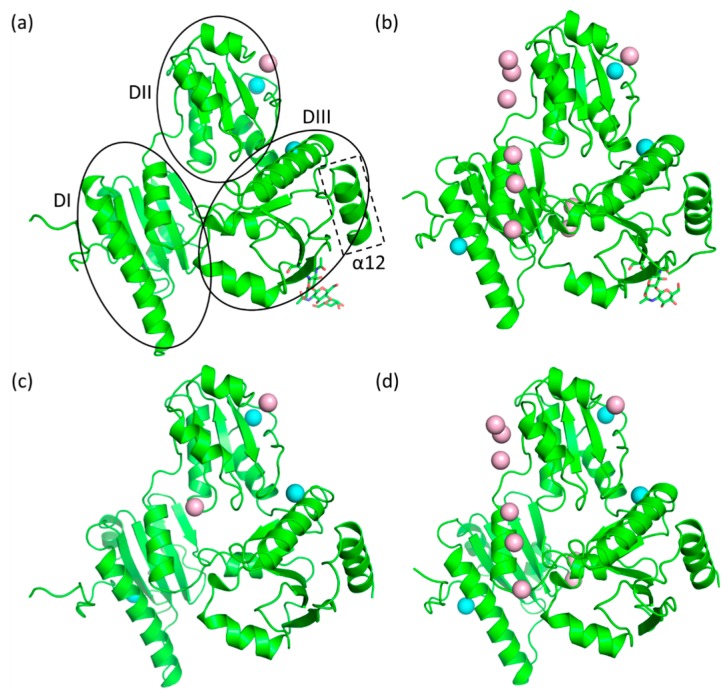
Crystal structures of (**a**) low-Ca^2+^ native bovine Casq1; (**b**) high-Ca^2+^ native bovine Casq1; (**c**) low-Ca^2+^ recombinant bovine Casq1 and (**d**) high-Ca^2+^ recombinant bovine Casq1. Cyan spheres represent Ca^2+^ bound at high-affinity sites, and pink spheres are Ca^2+^ bound at low-affinity sites. The glycosyl chains are represented as sticks. In (**a**), ovals and dotted lines indicate the boundary for the individual domains and C-terminal helix (α12).

Low-Ca^2+^ native bovine Casq1 crystallized in the P1 space group and contained four molecules in the unit cell (Figure 3a); the resolution of its diffraction data was 2.74 Å, whereas low-Ca^2+^ recombinant bovine Casq1 crystallized in the C222_1_ space group and contained one molecule in the asymmetric unit (Figure 3b), with a resolution of 1.88 Å. Both high-Ca^2+^ native (Figure 3c) and recombinant bovine Casq1 (Figure 3d) crystallized in the C222_1_ space group with three molecules in the asymmetric unit at resolutions of 2.60 and 2.14 Å, respectively.

**Figure 4:**
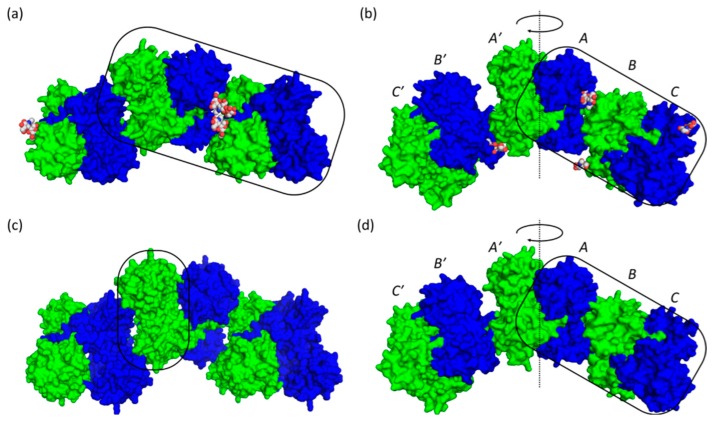
Lattice packing of (**a**) low-Ca^2+^ native bovine Casq1; (**b**) high-Ca^2+^ native bovine Casq1; (**c**) low-Ca^2+^ recombinant bovine Casq1 and (**d**) high-Ca^2+^ recombinant bovine Casq1. The pillboxes are approximate representations of the crystallographic asymmetric units. The positions of monomers A, B and C in the high-Ca^2+^ hexamers are labeled, along with their symmetry equivalents A′, B′ and C′, which are related by a two-fold rotation axis perpendicular to the long polymer axis, represented here as a dotted line and clockwise arrow within the front-to-front intermolecular interface formed between monomers A and A′. Individual Casq1 molecules were depicted with alternating colors of blue and green for easy view. The glycan moieties upon posttranslational modification were represented by the CPK model of grey (carbon atoms) and red (oxygen atoms) colors.

The lattice packing for both low-Ca^2+^ native (Figure 4a) and recombinant (Figure 4c) established a typical weakly-interacting linear polymer with D_2_ symmetry, a pattern also found in previous Casq structures [5,12,16,21]. On the other hand, the three molecules in the high-Ca^2+^ asymmetric unit of native (Figure 4c) and recombinant (Figure 4d) corresponded to one-half of a linear hexamer. The second half of the hexamer related to the asymmetric unit by a 180° rotation through the center of the hexamer, which was located within a front-to-front dimeric interface formed between monomer A and its symmetry-related equivalent, A′. Monomer B, which formed a dimer with monomer C through a front-to-front interaction, interacted with monomer A through a back-to-back interaction; monomers B′ and C′ interacted in a likewise fashion.

Each monomer in the low-Ca^2+^ structures of both native and recombinant Casq1 contained five Ca^2+^ ions, while the three monomers in the high-Ca^2+^ asymmetric units contained a total of 38 Ca^2+^ ions. In detail, the 38 Ca^2+^ ions in the asymmetric unit of three Casq1 monomers were distributed as follows: six ions were shared between A and A′; twelve Ca^2+^ ions were bound within the front-to-front interface of monomers B and C; and the remaining 20 ions were spread across the three monomers among their various structural Ca^2+^-binding sites and the high-capacity Ca^2+^ binding sites; therefore, the Casq1 hexamer contained 76 Ca^2+^ ions, with each dimer (A/A′, B/C and B′/C′) binding 12 Ca^2+^ ions cooperatively within their respective front-to-front interface and the remaining 40 Ca^2+^ ions bound non-cooperatively by structural and low-affinity, high-capacity sites.

In the case of low-Ca^2+^ native Casq1, the glycosyl groups on the four molecules in the crystallographic asymmetric unit modeled reliably into the corresponding electron density map as GlcNAc_2_ for two monomers, GlcNAc_2_Man_1_ for another monomer and GlcNAc_2_Man_2_ for the remaining monomer. In the high-Ca^2+^ native Casq1, the glycosyl groups were only modeled reliably up to GlcNAc_2_, as both the low resolution diffraction data and consequent low contour level of electron density were insufficient for building the remaining mannoses.

**Figure 5:**
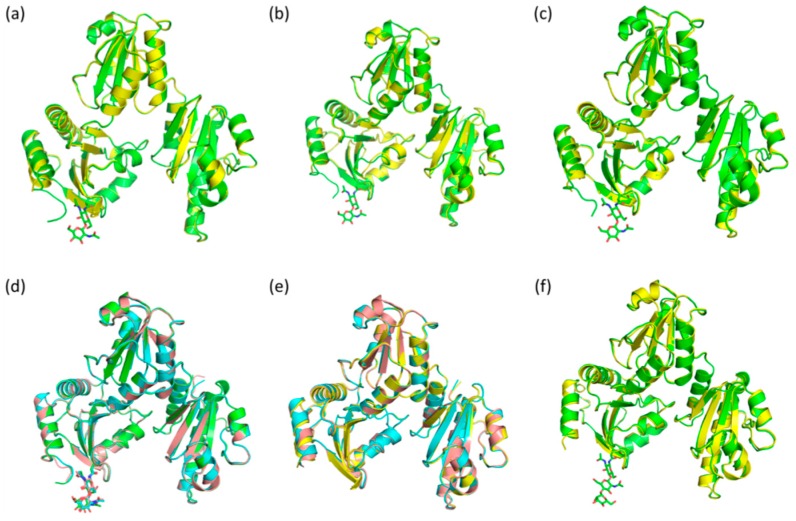
Cα atoms’ superposition of the high-Ca^2+^ native (green) and recombinant (yellow) bovine Casq1 (**a**) chain A, (**b**) chain B and (**c**) chain C; (**d**) Cα superposition of the high-Ca^2+^ native chains A (green), B (teal) and C (tan); (**e**) Cα atoms’ superposition of the high-Ca^2+^ native chains A (yellow), B (teal) and C (tan); (**f**) Cα atoms’ superposition of the low-Ca^2+^ native (green) and recombinant (yellow) bovine Casq1 structures. All superpositions were of residues Pro7 through Pro334, which correspond to Domains I through III, with the last secondary structural element of Domain III, α12 (residues Ser335 through Gly349) and residues 350 through 354 excluded.

To identify any possible glycosylation-dependent structural differences between native and recombinant calsequestrin, we superposed the Cα positions of Pro7 through Pro334 (i.e., Domains I to III, shown in Figure 3 (Figure 5 and Table 1).

Superposition of each monomer (A, B and C) of high-Ca^2+^ recombinant Casq1 onto the corresponding high-Ca^2+^ native Casq1 (A, B and C) had root mean square distances (r.m.s.ds) of 0.44, 0.41 and 0.25 Å, respectively (Figure 5a–c). Superposition of native A monomer on its own B and C (Figure 5d) had r.m.s.ds of 0.25 and 0.22 Å, respectively, whereas superposition of recombinant A monomer on its own B and C (Figure 5e) had r.m.s.ds of 0.56 and 0.40 Å, respectively. Superposition of low-Ca^2+^ recombinant Casq1 onto the four low-Ca^2+^ native monomers (Figure 5f) in the P1 unit cell had an average r.m.s.d of 0.40 Å.

**Figure 6:**
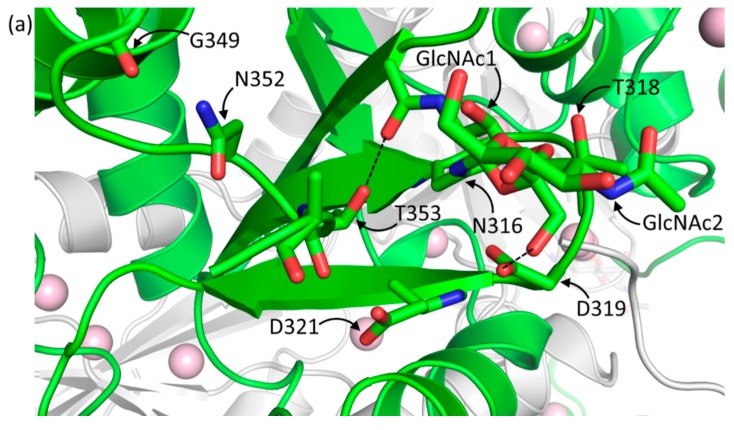

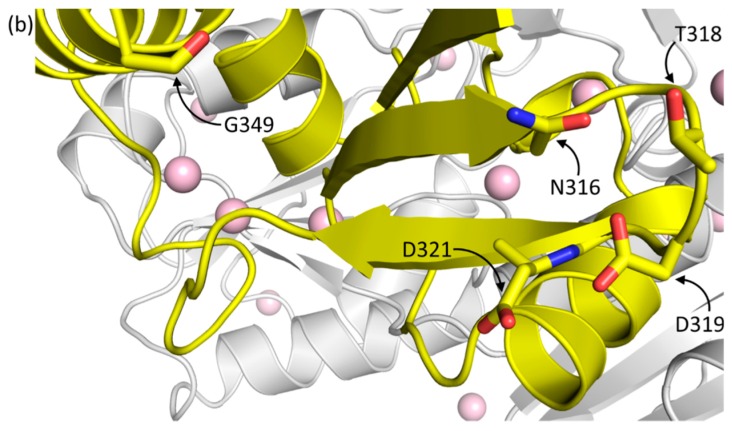
The glycosylation sites of high-Ca^2+^ (**a**) native bovine and (**b**) recombinant bovine. The labeled residues are involved in the stabilization of the glycosylation site and the Ca^2+^-dependent stretch of residues under high-Ca^2+^ conditions (Glu350 through Glu354). The dashed black lines indicate hydrogen bonds. The polypeptide backbone of native and recombinant bovine Casq1 are traced in green and yellow, respectively; and their respective dimeric partners are traced in gray. Green/yellow sticks are carbon atoms, red sticks are oxygen atoms, blue sticks are nitrogen atoms, and pink spheres are Ca^2+^ ions.

The similar r.m.s.d. values among native Casq1 monomers and the dissimilar values among recombinant monomers suggest that the glycosyl chains could help stabilize the conformation of individual Casq1 molecules. In high-Ca^2+^ native Casq1, the glycosyl chain of each monomer appears to stabilize a loop containing Glu350 through Glu354 by establishing a hydrogen bond between the *N*-acetyl group of the first *N*-acetylglucosamine and the hydroxyl group of Thr353 (Figure 6a). In addition to the glycosyl-mediated interaction, several other interactions stabilize the loop: (i) hydrogen bonds among the amide sidechain of Asn352, the backbone carbonyl of Gly349 and the backbone amide of Glu354; (ii) hydrophobic interactions between Ile351 and a hydrophobic pocket formed by Val323, Trp342 and Val346; (iii) hydrophobic interactions between the methyl group of Thr353 and a hydrophobic pocket formed by Ile249, Val314, C_β_ of Asn316 and Val323. Residues 350 through 354 in the crystal structure of high-Ca^2+^ recombinant Casq1 could not be modeled because they were not resolvable, which indicates that residues 350 through 354 are inherently flexible (Figure 6b).

**Figure 7:**
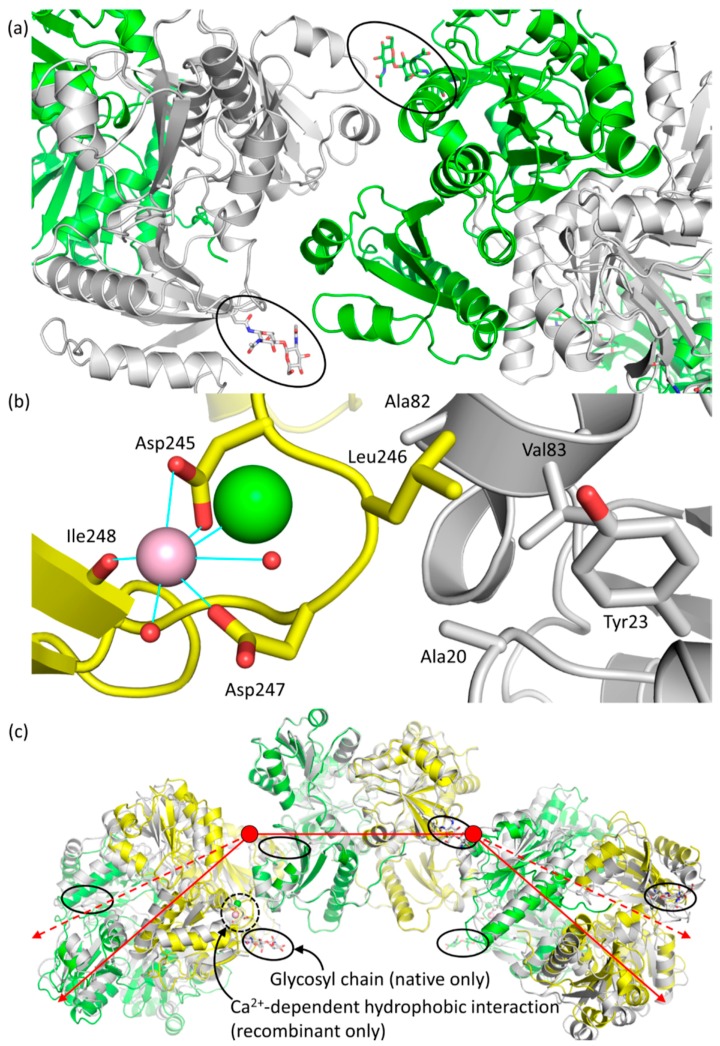
(**a**) An overhead view of a back-to-back interface showing the positioning of the glycosyl chains and the apparent steric constraints that they place on the polymer; (**b**) Close up of the Ca^2+^-dependent hydrophobic interaction seen in recombinant bovine Casq1. Relevant residues are labeled. The pink spheres represent Ca^2+^ ions and the green sphere represents a chloride ion, and the two small red spheres represent water molecules. Solid blue lines represent coordination bonds observed in the electron density map; (**c**) Least squares superposition of Cα atom in monomers A and A′ (central dimer) between their native hexameric form (from left to right, green, gray, green, gray, green and gray) and recombinant hexameric form (from left to right, gray, yellow, gray, yellow, gray and yellow). Glycosyl chains are circled with a black line, and the Ca^2+^-dependent hydrophobic interaction, only seen in high-Ca^2+^ recombinant, is circled with a black dotted line. The red lines are two-dimensional vector representations of the three dimers that compose the hexamer, with the dashed red lines indicating the deviation of recombinant Casq1 from native. In three-dimensional space and from a side-on perspective, each dimer of native Casq1 is rotated 18° with respect to the point at which two dimeric vectors meet (red circle) within their back-to-back interface, whereas recombinant Casq1 is rotated by only one.

Overall, when viewed from the top down, the glycosyl chains are oriented in a way that appears to hold the monomers in place (Figure 7a). This arrangement of the glycosyl chains is expected to prevent any lateral translation of dimers with respect to the long axis of the polymer, thereby promoting and/or stabilizing the linear polymer.

The larger structural variation among recombinant monomers, A, B and C, appeared to be due to a Leu246-mediated, Ca^2+^-dependent hydrophobic interaction that was only found in recombinant Casq1 upon exposure to a high Ca^2+^ concentration (Figure 7b). Residues Glu244 through His250 normally form a loop stabilized by hydrogen bonds, a salt bridge (between Arg223 and Glu244) and hydrophobic interactions of Leu246 with nearby residues. In monomer B of the high-Ca^2+^ recombinant Casq1, however, this loop was deformed upon binding of one Ca^2+^ ion by the carboxylate sidechains of Asp245 and Asp247 and the backbone carbonyl group of Ile249 (Figure 7b). Consequently, the bound Ca^2+^ ion displaced the sidechain of Leu246 and swung it outward across the back-to-back interface and into a hydrophobic pocket formed by Ala20, Tyr23, Ala82 and Val83 of chain A (Figure 7b). The Ca^2+^-dependent loop deformation also forced the backbone carbonyl of Gly248, which hydrogen bonds with the backbone amide of Val317 located in the middle of the N-linked glycosylation consensus sequence (^316^NVT^318^), downward against the glycosylation loop, which shifted the glycosylation loop accordingly. The shifted glycosylation loop then shifted α12, with which it interacts, outward towards the solvent. The net effect of the outward shift of α12 in monomer A and the Leu246-mediated, Ca^2+^-dependent hydrophobic interaction between monomers A and B was a break of the back-to-back symmetry. As a result, the recombinant hexamer was flatter when viewed from the side, in contrast to the bow shape of the native hexamer when viewed from the same angle (Figure 7c).

Due to the aforementioned effects, monomers A and B of high-Ca^2+^ native Casq1 do not superpose well with their equivalent of high-Ca^2+^ recombinant Casq1. However, monomers C of native and recombinant Casq1 displayed a smaller r.m.s.d of 0.23 Å, which indicates that they have similar conformations. Since monomer C and its symmetry equivalent, C′, are the terminal monomers of each respective hexamer and, as such, had only front-to-front interactions, it follows that there should only be a small difference due to the effect of glycosylation on Domain III.

### 2.4. Multi-Angle Light Scattering

**Figure 8:**
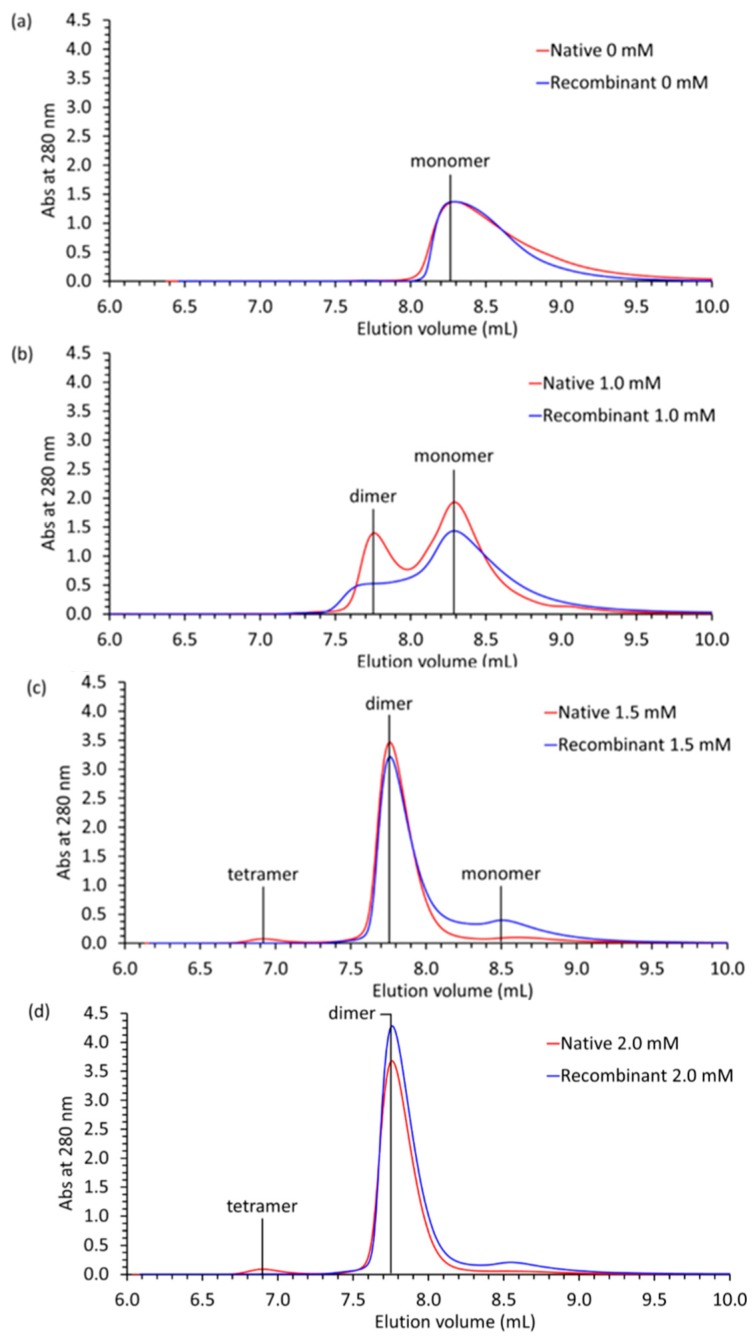
Native (red) and recombinant (blue) bovine Casq1 static light scattering/size-exclusion chromatography profiles, plotted as absorbance at 280 nm versus elution volume, in buffer containing either (**a**) 0, (**b**) 1.0, (**c**) 1.5 or (**d**) 2.0 mM Ca^2+^.

In order to measure a potential impact of glycosylation on the Ca^2+^-dependent polymerization property, the polymerization patterns for the wildtype Casq1 and recombinant Caq1 were compared by static light scattering. Tandem size-exclusion chromatography and multi-angle static light scattering analysis of native and recombinant bovine Casq1 (Figure 8) showed that native bovine Casq1 established higher-ordered polymers at lower concentrations than its recombinant counterpart did. Native and recombinant bovine Casq1 were present as monomers in buffer containing 0 mM Ca^2+^ (Figure 8a), but at 1 mM Ca^2+^ (Figure 8b), native bovine Casq1 was predominantly dimeric, whereas recombinant bovine Casq1 was predominantly monomeric. At 1.5 mM Ca^2+^ (Figure 8c), recombinant Casq1 showed a small presence of monomer. At 2 mM Ca^2+^ (Figure 8d), native Casq1 started to tetramerize, while recombinant Casq1 was solely dimeric.

## 3. Discussion

### 3.1. Evolution

**Figure 9:**
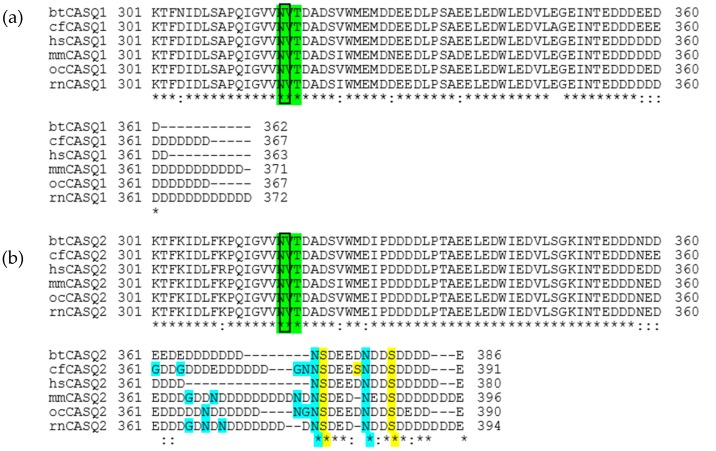
Casq1 and Casq2 sequence alignment of regions containing post-translational modifications (from Lys301 to the C-terminus). Asterisks indicate conserved residues, and colons indicate conserved substitutions. The eukaryotic N-linked glycosylation consensus sequence, conserved between Casq1 and Casq2, is highlighted in green, and the glycosylated residue, Asn316, is emphasized with a box. (**a**) Sequence alignment of bovine Casq1 (btCasq1), canine Casq1 (cfCasq1), human Casq1 (hsCasq1), mouse Casq1 (mmCasq1), rabbit Casq1 (ocCasq1) and rat Casq1 (rnCasq1); (**b**) sequence alignment of the cardiac isoforms of the same species. The serine residues boxed and highlighted in yellow are the putative phosphorylation sites in the Casq2 C-terminal tails (Glu354 to the C-terminal Glu). The residues highlighted in blue are the remaining non-acidic residues, which the Casq1 C-terminal tail does not contain. Canine Casq2 has a third serine residue, which may be a third phosphorylation site, in its C-terminal tail.

Human evolution diverged from its commonly-used laboratory test analogues, mice, rabbits and rats, to name a few, 61.5 million years ago [24]. Mice (genus *Mus*) and rats (genus *Rattus*), both of the Murinae subfamily of the family Muridae, are known to have split evolutionarily 11 million years ago [24,25], but despite the large separation in time, the Casq1 and Casq2 sequences of the common mouse and rat differ very little. This sequence conservation extends to other mammalian Casq1s and Casq2s (Table 2), the position of their glycosylation site at Asn316 and the number of phosphorylation sites in Casq2 (with the only exception thus far being canine cardiac, which has three phosphorylation sites) (Figure 9).

The sequence differences between Casq1 and Casq2 with maintained conservation among species extending to their post-translational modifications. As shown in Figure 1 and Figure 2, Casq2 from all tested animals features a higher degree of glycosylation than Casq1, with an average of six mannoses for Casq2, versus one mannose for Casq1. Casq2 is phosphorylated in all tested mammals, whereas Casq1 is not. Due to the high sequence identity and similar glycosylation/phosphorylation between human and bovine Casqs, bovine Casq1 and Casq2 could serve as reliable models for understanding the roles of those post-translational modifications in humans.

### 3.2. Glycosylation

**Figure 10:**
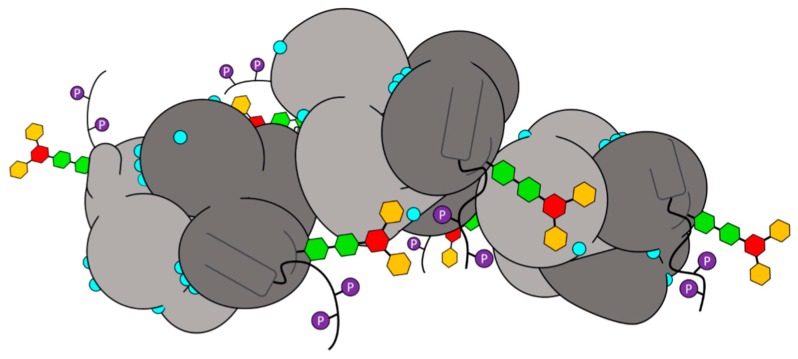
A representation of native human Casq2 based on the crystal structure of the native bovine Casq1 hexamer under high Ca^2+^ concentration. The alternating light and dark gray bodies represent the monomers in the hexamer, with the spherical domains representing Domains I, II and III, the triangular portions representing their N-terminal arms and cylindrical portions representing α12. Each glycan chain, GlcNAc_2_Man_3_ in this case, is represented by two green hexagons for the two *N*-acetylglucosamines, a red hexagon for the β-mannose and yellow for the two branched α-mannoses. The glycosylation loop is a dark gray semi-circle. The thick black lines pointing away from the polymer represent residues 350 through 354 and a hypothetical C-terminal tail. The two possible C-terminal phosphoryls in human Casq1 are purple circles with a white letter “P”. Cyan spheres mark the location of crystallographic Ca^2+^ ions that are visible from this perspective.

Glycosylation of Casq1 plays a role in both the initial step of polymerization by establishing the N-terminal arm-binding cavity necessary for the front-to-front interaction and in the following step of back-to-back interaction between two dimers. In all of these steps, glycosyl groups play a significant role not only in stabilizing the secondary and tertiary structures of Domain III in each monomer, but also in offering steric constraints to the polymer. As shown in Figure 7a, the glycosyl chains run parallel on both sides of the polymer, parallel to its long axis of the grown polymer, which likely prevents individual monomers from translating perpendicular to the polymer once being associated, which is reflected by the more homogenous conformation of native polymer (i.e., lower root-mean-squared deviation between monomers (Table 1)). These steric constraints thus serve like guiderails for individual monomers to establish a stable, linear polymer (Figure 10).

**Figure 11:**
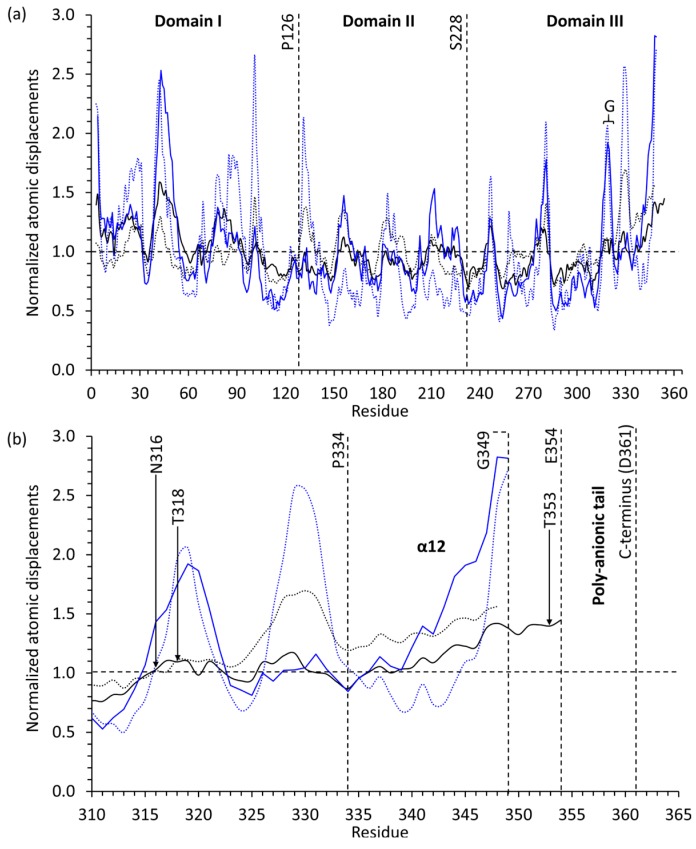
A plot of protein backbone atomic displacement parameters of the low-Ca^2+^ (black dashed lines for native, blue dashed lines for recombinant) and high-Ca^2+^ (black solid lines for native, and blue solid lines for recombinant) structures of native and recombinant bovine Casq1, normalized to their respective mean backbone atomic displacements and averaged for the asymmetric unit. (**a**) Dashed lines demarcate Domains I, II and III, and residue labels show the terminus of each domain. The letter G marks the location of the glycosylation loop; (**b**) close up of the 51 terminal residues. N316 is the N-linked glycosylation site; T318 is the terminal residue of the N-linked glycosylation sequence; and Thr353 is the residue seen to be hydrogen bonding with the first GlcNAc of high-Ca^2+^ native bovine Casq1.

The effect of these steric constraints are seen in the lower atomic displacements (B-factors) of the glycosylation loop of native Casq1 (Figure 11) together with the presence of well-defined electron density for residues 350 through 354.

These hypotheses are supported by the static light scattering profiles of native and recombinant bovine Casq1, which show that native bovine Casq1 (Figure 8a) undergoes monomer to dimer to tetramer transitions at lower Ca^2+^ concentrations than recombinant bovine Casq1 (Figure 8b). Thus, recombinant bovine, relative to its native form, is expected to require higher Ca^2+^ concentrations to polymerize in a Ca^2+^-dependent manner, as indicated by previous comparisons of static light scattering profiles and fractional occupancies [21].

In Casq2, our mass spectrometry data show the presence up to the full complement of nine mannoses found among tested animals, a substantially higher mannosylation than in Casq1. It is tempting to speculate that the presence of a longer glycosyl group is associated with the longer and more negatively-charged Casq2 C-terminal tail, as a greater enthalpic contribution is required to overcome the entropic cost for Ca^2+^-binding and polymerization. Indeed, and as shown by the high-Ca^2+^ structure of native bovine Casq1, the glycosyl chain helps to stabilize the expected orientation of the C-terminal tail towards the solvent; therefore, a bulkier six- to nine-mannose glycan chain may be more effective for orienting the longer Casq2 C-terminal tail. However, an excess of mannose residues between monomers would impede sterically their association into dimers (back-to-back interaction) [16]; the optimal glycan chain appears to be six mannose residues long, consistent with previous observations of heart failure correlated with Casq2 present predominantly as its NAcGlc_2_Man_8-9_ glycoform [26]. Under this interpretation of the role of glycosyls, too few mannose residues would also inhibit Casq2 polymerization, due to a loss of proper C-terminal tail orientation. Overall, altered polymerization due to improper glycosylation could prevent a proper function and localization of Casq.

### 3.3. Phosphorylation

Our mass spectra of the C-terminal peptide of native bovine, human and rat Casq2 showed the presence of up to two phosphorylation sites (Figure 2b), located in bovine Casq2 at Ser373 and Ser381; in human Casq2 at Ser366 and Ser374; and in rat Casq2 at Ser379 and Ser386 (Figure 9). Phosphorylation of Casq2 promotes α-helicity in its C-terminal tail and increases its Ca^2+^-binding capacity [17,27], making phosphorylation a potential regulator of the Ca^2+^-dependent responses of Casq2 in cardiac muscle. If so, an improper distribution of Casq2 with zero, one and two phosphoryls should have dire pathological consequences for an organism. In support of this hypothesis, defective or altered canine Casq2 phosphorylation is associated with several muscular complications, including heart failure [26,27,28,29,30,31,32]. Along with non-phosphorylated Casq2, our data showed the presence of singly- and doubly-phosphorylated Casq2. The dynamic or static level of phosphorylation of Casq2 could play different architectural roles in growing polymers [5]. For example, doubly-phosphorylated Casq2 could form a core linear polymer, mono-phosphorylated Casq2 participating in the more disordered off-branching tendrils and non-phosphorylated Casq2 as monomers with their tails facing toward solution capturing Ca^2+^ ions. Alternatively, Casq2 with phosphorylated C-terminal tails could be preferentially anchored near the RyR channel, as their more ordered conformation would have a higher affinity for continual attachment to triadin and junctin through a mechanism similar to that used by Casq1 [33].

### 3.4. Clinical Implications

Through this and previous works, we have shown that both Ca^2+^-dependent polymerization and Ca^2+^-binding capacity of Casq are modulated by glycosylation and phosphorylation. Those findings make it likely that such post-translational modifications are a tunable mechanism, operating in vivo. As any alteration of the unique characteristics of Casq could affect its Ca^2+^-binding capacity and the SR function, improper modification can produce pathophysiological consequences [16,17]. The exact glycosylation and phosphorylation patterns of both Casq1 (bovine, mouse and rat) and Casq2 (bovine, two humans and rat) established in this report provide compelling evidence of their similar nature across taxa and offer benchmarks to establish abnormal levels of post-translational modification.

Most CPVT2-related human Casq2 mutations are unable to form a proper Ca^2+^-dependent polymer [34]. In addition, certain small molecules with a significant affinity for binding to Casq, such as the anthracycline and phenothiazine derivatives, disrupt Ca^2+^-dependent Casq2 oligomerization and, eventually, SR function [35,36,37]. We propose that those Casq-binding drugs, CPVT2 mutations and altered post-translational modifications can have an additive or synergistic effect, leading to complications that are more serious when present in the same individual. Glycosylation may also affect the M87T human Casq1 mutant implicated as playing some role in malignant hyperthermia, due to the observed effect of the M87T mutation on the N-terminal arm, which terminates in a cavity formed, in part, by the glycosylation site.

## 4. Materials and Methods

### 4.1. Isolation of Cardiac and Skeletal Muscle Tissue

Samples of bovine, mouse and rat cardiac and skeletal muscle were obtained from Hereford cattle (local sources), Swiss mice (Washington State University colonies) and Sprague-Dawley rats (Simonsen Laboratories). All animal experiments were approved by the Institutional Animal Care and Use Committee (IACUC) (03951-001, 9/2/2009). Human cardiac muscle was obtained from Seoul National University Hospital of South Korea, under procedures approved by the Institutional Review Board of the Seoul National University Hospital. The heart tissues used in this study were from two patients (recipients) who received heart transplantation. Each heart was harvested after aortic and vena cava clamping along with cardiopulmonary bypass, and immediately, the prepared donor heart was transplanted. The first heart tissue was from a 59-year-old male, diagnosed with “enteropathy-associated T cell lymphoma”. The second heart tissue was from a 62-year-old male, diagnosed with “idiopathic dilated cardiomyopathy”. Both samples were obtained from the left ventricle, which is the site with abundant myocardium. The harvested heart tissues were washed in saline and instantly frozen in a deep freezer at −80 °C.

Left ventricular cardiac muscles were used to study bovine and human Casq2, and whole hearts were used to study mouse and rat Casq2. Chuck steak was used for bovine Casq1, whereas back and leg muscles were used to study mouse and rat Casq1.

### 4.2. Recombinant Bovine Casq1 Gene Synthesis

The bovine Casq1 gene (GenBank: AB277764.1) was synthesized by GenScript in pET28a(+) with *Xho*I and *Nco*I cut sites, following rare codon optimization (CGT for Arg, CGT for Leu, ATT for Ile and CCG for Pro) and transformed into Rosetta (DE3) pLysS *Escherichia coli*.

### 4.3. Purification of Native Casq1 and Casq2

Native Casq1 and Casq2 were extracted from the sarcoplasmic reticulum fractions of homogenized cardiac or muscle tissues from their respective organisms. Briefly, the muscle tissues were homogenized in a buffer containing 20 mM imidazole, 0.3 M sucrose, 0.15 M KCl and 0.05 g/dL NaN_3_, pH 7.4. The homogenates were clarified by centrifugation at 10,000× *g* for 15 min, followed by centrifugation at 100,000× *g* for 30 min in a Beckmann Ti 55.2 fixed-angle rotor to harvest the sarcoplasmic reticula. The sarcoplasmic reticulum pellets were suspended in a buffer containing 0.1 M Na_2_CO_3_, 10 mM EGTA, 0.5 M KCl and 0.05 g/dL NaN_3_ and incubated on ice for 10 min to extract Casq. After incubation, the carbonate extracts were centrifuged at 130,000× *g* for one hour. The carbonate extract supernatant was then loaded onto a phenyl sepharose column (Phenyl Sepharose 6 Fast Flow High Sub, GE Healthcare, Pittsburgh, PA, USA) equilibrated with phenyl sepharose wash buffer (20 mM MOPS, 1 mM EGTA, 0.5 M NaCl, 0.05 g/dL NaN_3_, pH 7.2). After washing the column extensively with phenyl sepharose wash buffer, Casq was eluted using phenyl sepharose wash buffer containing 10 mM CaCl_2_. The eluates were then buffer exchanged into anion exchange Buffer A (20 mM Tris, pH 8.5, 0.05 g/dL NaN_3_) and loaded on to a MonoQ column (GE Healthcare). Contaminating proteins were eluted from the column using a stepwise gradient from 0% to 25% Buffer B (20 mM Tris, 2 M NaCl, 0.05 g/dL NaN_3_, pH 8.5), followed by elution of Casq using a stepwise gradient from 25% to 28.5% Buffer B. The purified proteins were then pooled, concentrated, dialyzed overnight against double reverse osmosis H_2_O to remove trace Ca^2+^ and buffer exchanged into buffers suitable for their given purpose.

### 4.4. Purification of Recombinant Bovine Casq1

Recombinant bovine Casq1 was purified using previously-established protocols [21]. Fractions containing the purified proteins were then prepared for crystallization and experiments using the same protocol used for the native Casqs.

### 4.5. Mass Spectrometry

For the purpose of fragmentation, protein solutions were allowed to react for 2 hours in the dark and at room temperature with a solution consisting of 2 M Cyanogen bromide (CNBr), 10 mM Caesium iodide (CsI) and 70% (*v*/*v*) trifluoroacetic acid (TFA). The samples were then dried, suspended in water and loaded into a NuTip C8 tip (Glygen Corporation, Columbia, MD, USA). The tip was washed with 0.1% (*v*/*v*) TFA in water, followed by eluting Casq from the tip with 100% acetonitrile. Elutions were then mixed with an equal volume of 10 mg·mL^−1^ sinapic acid in 50% (*v*/*v*) acetonitrile with 0.1% (*v*/*v*) TFA, spotted onto a sample plate containing dry matrix and allowed to dry. Phosphopeptides were enriched from the total peptide solution using the NuTip TiO_2_ + ZrO_2_ tip (Glygen Corporation) and manufacturer protocols. The eluted peptides were acidified with TFA, dried, suspended in sinapic matrix and spotted onto a sample plate containing dried matrix. The spectra for the peptides were collected using a 4800 MALDI TOF/TOF Analyzer (Applied Biosystems, Carlsbad, CA, USA). Mass spectra were collected in positive and negative linear and reflector modes, and the best spectra were chosen based on signal-to-noise ratio. Specifically, the best Casq1 spectra were collected in negative reflector mode, whereas the best Casq2 spectra were collected in linear positive mode, except for the glycosylation of bovine Casq2 and one of the human hearts. The mass of Casq was calibrated internally using the +1 and +2 BSA ions.

### 4.6. Multi-Angle Light Scattering

Native and recombinant bovine Casq1 at 50 µM in static-light scattering running buffer (20 mM MOPS, 300 mM KCl, 0.05 g/dL NaN_3_, pH 7.2) containing either 0, 1, 1.5 or 2 mM CaCl_2_ were injected onto a Yarra 3u SEC-2000 size-exclusion column (Phenomenex, Torrance, CA, USA) equilibrated with the same buffer. The presence of Casq1 was monitored at 280 nm in tandem with a DAWN EOS multi-angle light scattering machine (Wyatt Technology, Goleta, CA, USA) for measuring scattering intensities.

### 4.7. Crystallization

Native and recombinant bovine Casq1 (0.3 mM in 20 mM HEPES, 0.5 M NaCl, 0.05 g/dL NaN_3_, pH 7.0) were crystallized using the hanging drop vapor diffusion method at 4 °C. Protein solutions were mixed with an equal volume of crystallization buffer (0.1 M HEPES, 0.2 M NaCl, 27.5% (*v*/*v*) 2-methyl-2,4-pentanediol, pH 7.0), and crystals formed overnight. To obtain the high-Ca^2+^ native and recombinant bovine Casq1 structures, each protein was incubated with 5 mM Ca^2+^ prior to mixing with crystallization solution. Crystallographic data were collected at the Advanced Light Source (Beamlines 8.2.1 and 8.2.2) and reduced and scaled using HKL2000. The low-Ca^2+^ native and recombinant bovine Casq1 structures were solved by molecular replacement PHENIX [38] using the deposited coordinates of native rabbit Casq1 (PDB ID: 3TRQ) and recombinant rabbit Casq1 (PDB ID: 3TRP), respectively. The high-Ca^2+^ recombinant bovine Casq1 structure was solved by molecular replacement using the low-Ca^2+^ native bovine Casq1 structure. After rebuilding the low-Ca^2+^ structure to its high-Ca^2+^ form, a high-Ca^2+^ recombinant bovine monomer was used to solve the high-Ca^2+^ native bovine Casq1 structure by molecular replacement. Iterative model adjustment and refinement were completed using COOT [39] and PHENIX. TLS (translation/libration/screw) groups used in refinement were determined by the TLS Motion Determination (TLSMD) web server [40]. The crystallographic coordinates and structure factors for low-Ca^2+^ native (PDB ID: 5KN0), low-Ca^2+^ recombinant (PDB ID: 5KN3), high-Ca^2+^ native (PDB ID: 5KN2) and high-Ca^2+^ recombinant (PDB ID: 5KN1) have been deposited in the Protein Data Bank. Refinement statistics are listed in Table 3.

## Figures and Tables

**Table 1:** Root-mean-squared deviation (in Å) for Cα least-squares superposition of Pro7 through Pro334 between the high-Ca^2+^ structures of native and recombinant bovine Casq1.

Protein and Chain Identity		High-Ca^2+^ Native			High-Ca^2+^ Recombinant		
		Chain A	Chain B	Chain C	Chain A	Chain B	Chain C
High-Ca^2+^ recombinant	Chain C	0.245	0.269	0.234	0.401	0.391	0
	Chain B	0.380	0.412	0.399	0.535	0	–
	Chain A	0.438	0.464	0.435	0	–	–
High-Ca^2+^ native	Chain C	0.205	0.228	0	–	–	–
	Chain B	0.234	0	–	–	–	–
	Chain A	0	–	–	–	–	–

**Table 2:** Casq1 and Casq2 percent identity and similarity table.

Protein	btCasq2	cfCasq2	hsCasq2	mmCasq2	ocCasq2	rnCasq2	rnCasq1	ocCasq1	mmCasq1	hsCasq1	cfCasq1
btCasq1	69.53	69.81	70.08	68.70	68.98	68.70	95.84	94.74	95.01	94.18	95.84
	(93.35)	(92.80)	(93.35)	(92.80)	(91.97)	(92.24)	(99.45)	(98.89)	(99.45)	(99.17)	(99.17)
cfCasq1	69.21	70.30	69.75	68.39	68.94	68.39	96.19	96.73	95.37	96.41	–
	(93.73)	(93.19)	(94.01)	(92.64)	(92.64)	(92.64)	(99.73)	(99.18)	(99.73)	(99.45)	–
hsCasq1	68.51	68.51	69.06	66.57	67.68	66.85	97.51	96.41	96.41	–	–
	(93.37)	(93.09)	(93.65)	(92.54)	(92.27)	(92.54)	(100)	(99.17)	(99.72)	–	–
mmCasq1	69.00	68.73	68.46	67.39	68.19	66.85	99.19	95.37	–	–	–
	(93.90)	(93.26)	(94.07)	(92.72)	(92.45)	(92.72)	(100)	(99.46)	–	–	–
ocCasq1	69.48	70.03	69.75	68.94	69.21	66.21	96.19	–	–	–	–
	(94.01)	(93.46)	(94.28)	(92.92)	(92.92)	(90.19)	(99.46)	–	–	–	–
rnCasq1	69.35	69.35	69.09	68.01	68.82	67.47	–	–	–	–	–
	(93.82)	(93.28)	(94.09)	(92.74)	(97.24)	(92.74)	–	–	–	–	–
rnCasq2	90.41	91.30	91.58	97.21	92.05	–	–	–	–	–	–
	(98.96)	(97.70)	(98.42)	(99.49)	(97.69)	–	–	–	–	–	–
ocCasq2	94.04	93.85	93.95	91.28	–	–	–	–	–	–	–
	(98.45)	(97.95)	(98.16)	(97.69)	–	–	–	–	–	–	–
mmCasq2	89.12	90.28	90.53	–	–	–	–	–	–	–	–
	(98.70)	(97.70)	(98.42)	–	–	–	–	–	–	–	–
hsCasq2	95.26	93.68	–	–	–	–	–	–	–	–	–
	(99.47)	(98.42)	–	–	–	–	–	–	–	–	–
cfCasq2	95.60	–	–	–	–	–	–	–	–	–	–
	(98.96)	–	–	–	–	–	–	–	–	–	–

**Table 3:** X-ray data collection and refinement statistics. TLS, translation/libration/screw.

Protein	Low-Ca^2+^ Native Bovine Casq1	High-Ca^2+^ Native Bovine Casq1	Low-Ca^2+^ Recombinant Bovine Casq1	High-Ca^2+^ Recombinant Bovine Casq1
PDB ID	5KN0	5KN2	5KN3	5KN1
Data collection	–	–	–	–
Space group	P1	C222_1_	C222_1_	C222_1_
Cell dimensions	–	–	–	–
*a*, *b*, *c* (Å)	60.342, 92.994, 101.849	130.363, 169.194, 155.477	59.393, 146.06, 110.34	135.669, 165.604, 156.626
*α*, *β*, *γ* (°)	71.122, 84.574, 73.485	90.0, 90.0, 90.0	90.0, 90.0, 90.0	90.0, 90.0, 90.0
Resolution (Å)	49.66–2.73 (2.83–2.73)	42.95–2.60 (2.70–2.60)	49.20–1.85 (1.92–1.85)	43.63–2.14 (2.21–2.14)
*R_merge_*	0.0318 (0.3289)	0.0633 (1.416)	0.0698 (0.8201)	0.0922 (0.8878)
*<I>/<σI>*	14.84 (2.55)	13.25 (1.34)	13.40 (2.72)	27.16 (5.0)
Completeness (%)	0.98 (0.94)	0.99 (0.97)	0.100 (0.97)	0.99 (0.94)
Multiplicity	2.0 (1.9)	7.3 (7.4)	7.1 (6.6)	7.1 (6.8)
Refinement	–	–	–	–
Resolution	49.7–2.73 (2.83–2.73)	42.95–2.60 (2.70–2.60)	49.20–1.85 (1.92–1.85)	43.63–2.14 (2.21–2.14)
Unique reflections	52,550 (5026)	52,807 (5112)	41,309 (3984)	96,658 (9078)
*R_work_/R_free_*	0.1970/0.241 (0.3474/0.3834)	0.2056/0.2411 (0.3302/0.3536)	0.1833/0.2106 (0.2936/0.3201)	0.1836/0.2011 (0.2429/0.2594)
Number of atoms	–	–	–	–
Macromolecules	11,172	8456	2827	8466
Ion	19	39	5	40
Ligand	175	84	32	33
Water molecules	33	5	336	461
R.m.s deviations	–	–	–	–
Bond lengths (Å)	0.005	0.005	0.003	0.005
Bond angles (°)	0.71	0.84	0.57	0.69
Ramachandrans	–	–	–	–
Favored (%)	97.6	98.0	98.6	98.0
Outliers (%)	0	0	0	0
Clashscore	1.59	1.03	2.70	1.82
B-factors	–	–	–	–
Protein	76.71	108.25	43.83	59.42
Ligand/Ion	100.15	139.00	69.90	74.69
Water	59.89	67.90	44.92	51.10
TLS groups	27	13	20	60
